# 3,5-Dichloro-*N*-(2-methyl­but-3-yn-2-yl)benzamide

**DOI:** 10.1107/S1600536809051228

**Published:** 2009-12-04

**Authors:** Zong-Ling Ru, Guo-Xi Wang

**Affiliations:** a Department of Chemical & Environmental Engineering, Anyang Institute of Technology, Anyang 455000, People’s Republic of China

## Abstract

In the title compound, C_12_H_11_Cl_2_NO, the amide group is twisted by a dihedral angle of 31.98 (2)° with respect to the benzene ring. In the crystal structure, mol­ecules are linked *via* N—H⋯O hydrogen bonds, forming one-dimensional supra­molecular chains.

## Related literature

For the chemistry of halogenated aromatic amide derivatives, see: Cirilli *et al.* (1997 ▶).
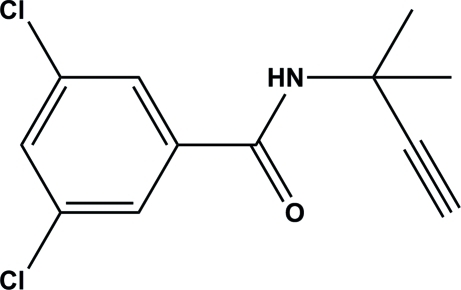


## Experimental

### 

#### Crystal data

C_12_H_11_Cl_2_NO
*M* *_r_* = 256.12
Monoclinic, 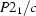

*a* = 12.227 (2) Å
*b* = 10.898 (2) Å
*c* = 10.170 (2) Å
β = 111.08 (3)°
*V* = 1264.5 (4) Å^3^
*Z* = 4
Mo *K*α radiation
μ = 0.49 mm^−1^
*T* = 298 K
0.4 × 0.35 × 0.2 mm

#### Data collection

Rigaku Mercury2 diffractometer
Absorption correction: multi-scan (*CrystalClear*; Rigaku, 2005 ▶) *T*_min_ = 0.881, *T*_max_ = 0.940
12803 measured reflections
2890 independent reflections
2308 reflections with *I* > 2σ(*I*)
*R*_int_ = 0.031

#### Refinement

*R*[*F* ^2^ > 2σ(*F* ^2^)] = 0.043
*wR*(*F* ^2^) = 0.103
*S* = 1.07
2890 reflections
147 parameters
1 restraint
H-atom parameters constrained
Δρ_max_ = 0.28 e Å^−3^
Δρ_min_ = −0.29 e Å^−3^

### 

Data collection: *CrystalClear* (Rigaku, 2005 ▶); cell refinement: *CrystalClear*; data reduction: *CrystalClear*; program(s) used to solve structure: *SHELXTL* (Sheldrick, 2008 ▶); program(s) used to refine structure: *SHELXTL*; molecular graphics: *SHELXTL*; software used to prepare material for publication: *SHELXTL*.

## Supplementary Material

Crystal structure: contains datablocks I, global. DOI: 10.1107/S1600536809051228/xu2695sup1.cif

Structure factors: contains datablocks I. DOI: 10.1107/S1600536809051228/xu2695Isup2.hkl

Additional supplementary materials:  crystallographic information; 3D view; checkCIF report

## Figures and Tables

**Table 1:** Hydrogen-bond geometry (Å, °)

*D*—H⋯*A*	*D*—H	H⋯*A*	*D*⋯*A*	*D*—H⋯*A*
N1—H1*B*⋯O1^i^	0.86	2.21	3.051 (3)	168

Symmetry code: (i) 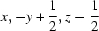.

**Fig. 1.**
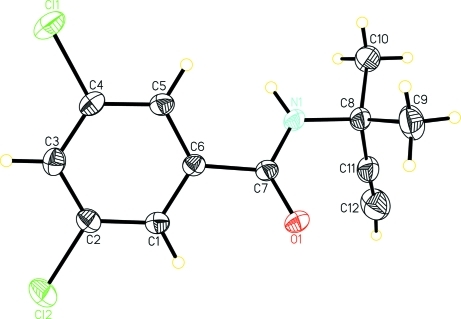
A view of the title compound with the atomic numbering scheme. Displacement ellipsoids were drawn at the 30% probability level.

## supplementary crystallographic information

### Comment

Halogenated aromatic amide derivatives are an important class of chemical raw materials, which have found wide range of applications in agriculture as herbicides, in medicine as drugs, in coordination chemistry as ligand, and which are also used in industry. Recently, a series of halogenated aromatic amide compounds have been reported (Cirilli *et al.*, 1997). As an extension of these work on the structural characterization, we report here the crystal structure of the title compound 3,5-dichloro-*N*-(2-methylbut-3-yn-2-yl)benzamide.

The crystal data show that in the title compound (Fig. 1), the amide group is rotated by 31.98 (2)° out of the plane of the benzene ring. All the bond length are within the normal range. The crystal packing is stabilized by N—H···O hydrogen bonds to form an infinite one-dimensional chain parallel to the *c* axis (Table 1).

### Experimental

The purchased 3,5-dichloro-*N*-(2-methylbut-3-yn-2-yl)benzamide (3 mmol, 768 mg) was dissolved in chloroform (20 ml) and evaporated in the air, single crystals of the compound suitable for X-ray analysis were obtained from the solution.

### Refinement

The acetylene H atom was located in a difference Fourier map and refined as riding in as-found relative position with U_iso_(H) = 1.2U_eq_(C). Other H atoms were placed in calculated positions and refined in riding mode with C–H = 0.93 (aromatic), 0.96 Å (methyl) and N–H = 0.86 Å, U_iso_(H) = 1.5U_eq_(C) for methyl H atoms and 1.2U_eq_(C,N) for the others.

### Crystal data

**Table:**

C_12_H_11_Cl_2_NO	*F*(000) = 528
*M**_r_* = 256.12	*D*_x_ = 1.345 Mg m^−^^3^
Monoclinic, *P*2_1_/*c*	Mo *K*α radiation, λ = 0.71073 Å
Hall symbol: -P 2ybc	Cell parameters from 2308 reflections
*a* = 12.227 (2) Å	θ = 3.6–27.5°
*b* = 10.898 (2) Å	µ = 0.49 mm^−^^1^
*c* = 10.170 (2) Å	*T* = 298 K
β = 111.08 (3)°	Block, colourless
*V* = 1264.5 (4) Å^3^	0.4 × 0.35 × 0.2 mm
*Z* = 4	

### Data collection

**Table:**

Rigaku Mercury2 diffractometer	2890 independent reflections
Radiation source: fine-focus sealed tube	2308 reflections with *I* > 2σ(*I*)
graphite	*R*_int_ = 0.031
Detector resolution: 13.6612 pixels mm^-1^	θ_max_ = 27.5°, θ_min_ = 3.6°
ω scans	*h* = −15→15
Absorption correction: multi-scan (*CrystalClear*; Rigaku, 2005)	*k* = −14→14
*T*_min_ = 0.881, *T*_max_ = 0.940	*l* = −13→13
12803 measured reflections	

### Refinement

**Table:**

Refinement on *F*^2^	Primary atom site location: structure-invariant direct methods
Least-squares matrix: full	Secondary atom site location: difference Fourier map
*R*[*F*^2^ > 2σ(*F*^2^)] = 0.043	Hydrogen site location: inferred from neighbouring sites
*wR*(*F*^2^) = 0.103	H-atom parameters constrained
*S* = 1.07	*w* = 1/[σ^2^(*F*_o_^2^) + (0.0378*P*)^2^ + 0.5464*P*] where *P* = (*F*_o_^2^ + 2*F*_c_^2^)/3
2890 reflections	(Δ/σ)_max_ < 0.001
147 parameters	Δρ_max_ = 0.28 e Å^−^^3^
1 restraint	Δρ_min_ = −0.29 e Å^−^^3^

### Special details

**Table:**

Geometry. All e.s.d.'s (except the e.s.d. in the dihedral angle between two l.s. planes) are estimated using the full covariance matrix. The cell e.s.d.'s are taken into account individually in the estimation of e.s.d.'s in distances, angles and torsion angles; correlations between e.s.d.'s in cell parameters are only used when they are defined by crystal symmetry. An approximate (isotropic) treatment of cell e.s.d.'s is used for estimating e.s.d.'s involving l.s. planes.
Refinement. Refinement of *F*^2^ against ALL reflections. The weighted *R*-factor *wR* and goodness of fit *S* are based on *F*^2^, conventional *R*-factors *R* are based on *F*, with *F* set to zero for negative *F*^2^. The threshold expression of *F*^2^ > σ(*F*^2^) is used only for calculating *R*-factors(gt) *etc*. and is not relevant to the choice of reflections for refinement. *R*-factors based on *F*^2^ are statistically about twice as large as those based on *F*, and *R*- factors based on ALL data will be even larger.

### Fractional atomic coordinates and isotropic or equivalent isotropic displacement parameters (Å^2^)

**Table:**

	*x*	*y*	*z*	*U*_iso_*/*U*_eq_	
Cl1	0.50021 (8)	0.45984 (9)	0.16860 (9)	0.0697 (3)	
Cl2	0.35913 (8)	0.74552 (7)	0.50663 (10)	0.0644 (3)	
O1	0.20562 (18)	0.29208 (18)	0.52618 (18)	0.0477 (5)	
C1	0.2939 (2)	0.5093 (2)	0.4506 (3)	0.0376 (6)	
H1A	0.2532	0.5195	0.5115	0.045*	
C7	0.2183 (2)	0.2973 (2)	0.4121 (2)	0.0349 (5)	
C5	0.3539 (2)	0.3825 (2)	0.2953 (3)	0.0387 (6)	
H5A	0.3524	0.3081	0.2499	0.046*	
C6	0.2908 (2)	0.3977 (2)	0.3837 (2)	0.0334 (5)	
N1	0.1679 (2)	0.2190 (2)	0.3067 (2)	0.0420 (5)	
H1B	0.1830	0.2271	0.2308	0.050*	
C3	0.4220 (2)	0.5918 (3)	0.3399 (3)	0.0433 (6)	
H3A	0.4660	0.6565	0.3253	0.052*	
C2	0.3579 (2)	0.6049 (2)	0.4260 (3)	0.0399 (6)	
C4	0.4190 (2)	0.4799 (3)	0.2760 (3)	0.0415 (6)	
C8	0.0882 (3)	0.1195 (2)	0.3123 (3)	0.0444 (6)	
C11	−0.0141 (3)	0.1721 (3)	0.3361 (3)	0.0543 (8)	
C10	0.0456 (3)	0.0569 (3)	0.1680 (3)	0.0680 (10)	
H10A	0.0071	0.1160	0.0963	0.102*	
H10B	−0.0083	−0.0075	0.1667	0.102*	
H10C	0.1114	0.0228	0.1503	0.102*	
C12	−0.0996 (4)	0.2091 (4)	0.3488 (5)	0.0849 (12)	
H12	−0.1710	0.2530	0.3603	0.102*	
C9	0.1506 (3)	0.0267 (3)	0.4266 (4)	0.0707 (10)	
H9A	0.1726	0.0653	0.5173	0.106*	
H9B	0.2194	−0.0029	0.4124	0.106*	
H9C	0.0990	−0.0409	0.4222	0.106*	

### Atomic displacement parameters (Å^2^)

**Table:**

	*U*^11^	*U*^22^	*U*^33^	*U*^12^	*U*^13^	*U*^23^
Cl1	0.0835 (6)	0.0804 (6)	0.0687 (5)	−0.0018 (5)	0.0559 (5)	0.0028 (4)
Cl2	0.0809 (6)	0.0369 (4)	0.0803 (6)	−0.0041 (4)	0.0351 (5)	−0.0126 (4)
O1	0.0674 (13)	0.0510 (11)	0.0302 (9)	−0.0099 (10)	0.0240 (9)	−0.0033 (8)
C1	0.0375 (14)	0.0413 (14)	0.0341 (13)	0.0005 (11)	0.0129 (11)	−0.0027 (10)
C7	0.0394 (13)	0.0373 (13)	0.0280 (12)	−0.0003 (10)	0.0119 (10)	−0.0009 (10)
C5	0.0443 (14)	0.0413 (14)	0.0314 (12)	−0.0004 (11)	0.0147 (11)	−0.0025 (10)
C6	0.0329 (12)	0.0388 (13)	0.0259 (11)	−0.0012 (10)	0.0074 (9)	−0.0004 (10)
N1	0.0537 (14)	0.0465 (13)	0.0299 (11)	−0.0158 (10)	0.0199 (10)	−0.0063 (9)
C3	0.0435 (15)	0.0432 (15)	0.0422 (14)	−0.0038 (12)	0.0143 (12)	0.0073 (12)
C2	0.0427 (14)	0.0345 (13)	0.0397 (14)	0.0020 (11)	0.0114 (11)	−0.0016 (10)
C4	0.0428 (15)	0.0511 (16)	0.0343 (13)	0.0021 (12)	0.0186 (11)	0.0052 (11)
C8	0.0569 (17)	0.0405 (15)	0.0376 (14)	−0.0128 (12)	0.0193 (13)	−0.0036 (11)
C11	0.060 (2)	0.0516 (18)	0.0552 (18)	−0.0166 (15)	0.0249 (15)	−0.0028 (14)
C10	0.091 (3)	0.066 (2)	0.0528 (19)	−0.0371 (19)	0.0325 (18)	−0.0231 (16)
C12	0.070 (3)	0.078 (3)	0.114 (3)	−0.013 (2)	0.042 (2)	−0.013 (2)
C9	0.089 (3)	0.0462 (19)	0.070 (2)	−0.0042 (17)	0.020 (2)	0.0103 (16)

### Geometric parameters (Å, °)

**Table:**

Cl1—C4	1.734 (3)	C3—C2	1.377 (4)
Cl2—C2	1.736 (3)	C3—H3A	0.9300
O1—C7	1.226 (3)	C8—C11	1.472 (4)
C1—C2	1.380 (4)	C8—C9	1.522 (4)
C1—C6	1.387 (3)	C8—C10	1.531 (4)
C1—H1A	0.9300	C11—C12	1.171 (5)
C7—N1	1.335 (3)	C10—H10A	0.9600
C7—C6	1.500 (3)	C10—H10B	0.9600
C5—C4	1.383 (4)	C10—H10C	0.9600
C5—C6	1.390 (3)	C12—H12	1.0386
C5—H5A	0.9300	C9—H9A	0.9600
N1—C8	1.473 (3)	C9—H9B	0.9600
N1—H1B	0.8600	C9—H9C	0.9600
C3—C4	1.375 (4)		
			
C2—C1—C6	119.3 (2)	C3—C4—Cl1	119.0 (2)
C2—C1—H1A	120.3	C5—C4—Cl1	118.8 (2)
C6—C1—H1A	120.3	C11—C8—N1	109.4 (2)
O1—C7—N1	123.5 (2)	C11—C8—C9	110.8 (3)
O1—C7—C6	120.1 (2)	N1—C8—C9	111.2 (2)
N1—C7—C6	116.5 (2)	C11—C8—C10	108.4 (3)
C4—C5—C6	118.8 (2)	N1—C8—C10	107.0 (2)
C4—C5—H5A	120.6	C9—C8—C10	109.9 (3)
C6—C5—H5A	120.6	C12—C11—C8	175.9 (4)
C1—C6—C5	119.9 (2)	C8—C10—H10A	109.5
C1—C6—C7	117.3 (2)	C8—C10—H10B	109.5
C5—C6—C7	122.8 (2)	H10A—C10—H10B	109.5
C7—N1—C8	124.0 (2)	C8—C10—H10C	109.5
C7—N1—H1B	118.0	H10A—C10—H10C	109.5
C8—N1—H1B	118.0	H10B—C10—H10C	109.5
C4—C3—C2	117.9 (2)	C11—C12—H12	172.7
C4—C3—H3A	121.0	C8—C9—H9A	109.5
C2—C3—H3A	121.0	C8—C9—H9B	109.5
C3—C2—C1	121.8 (2)	H9A—C9—H9B	109.5
C3—C2—Cl2	118.9 (2)	C8—C9—H9C	109.5
C1—C2—Cl2	119.3 (2)	H9A—C9—H9C	109.5
C3—C4—C5	122.2 (2)	H9B—C9—H9C	109.5
			
C2—C1—C6—C5	1.4 (4)	C4—C3—C2—Cl2	−179.4 (2)
C2—C1—C6—C7	−179.0 (2)	C6—C1—C2—C3	−2.0 (4)
C4—C5—C6—C1	0.0 (4)	C6—C1—C2—Cl2	178.54 (19)
C4—C5—C6—C7	−179.6 (2)	C2—C3—C4—C5	0.3 (4)
O1—C7—C6—C1	−30.5 (4)	C2—C3—C4—Cl1	−179.2 (2)
N1—C7—C6—C1	147.8 (2)	C6—C5—C4—C3	−0.9 (4)
O1—C7—C6—C5	149.1 (2)	C6—C5—C4—Cl1	178.65 (19)
N1—C7—C6—C5	−32.6 (4)	C7—N1—C8—C11	59.8 (4)
O1—C7—N1—C8	2.1 (4)	C7—N1—C8—C9	−63.0 (4)
C6—C7—N1—C8	−176.2 (2)	C7—N1—C8—C10	177.0 (3)
C4—C3—C2—C1	1.1 (4)		

### Hydrogen-bond geometry (Å, °)

**Table:**

*D*—H···*A*	*D*—H	H···*A*	*D*···*A*	*D*—H···*A*
N1—H1B···O1^i^	0.86	2.21	3.051 (3)	168

Symmetry codes: (i) *x*, −*y*+1/2, *z*−1/2.
